# Venovenous Extracorporeal Membrane Oxygenation in Intractable Pulmonary Insufficiency: Practical Issues and Future Directions

**DOI:** 10.1155/2016/9367464

**Published:** 2016-04-05

**Authors:** T. S. R. Delnoij, R. Driessen, A. S. Sharma, E. A. Bouman, U. Strauch, P. M. Roekaerts

**Affiliations:** ^1^Department of Cardiology, Maastricht University Medical Center, 6202 AZ Maastricht, Netherlands; ^2^Department of Intensive Care, Maastricht University Medical Center, 6202 AZ Maastricht, Netherlands; ^3^Department of Cardiothoracic Surgery, Maastricht University Medical Center, 6202 AZ Maastricht, Netherlands; ^4^Department of Anesthesiology, Maastricht University Medical Center, 6202 AZ Maastricht, Netherlands

## Abstract

Venovenous extracorporeal membrane oxygenation (vv-ECMO) is a highly invasive method for organ support that is gaining in popularity due to recent technical advances and its successful application in the recent H1N1 epidemic. Although running a vv-ECMO program is potentially feasible for many hospitals, there are many theoretical concepts and practical issues that merit attention and require expertise. In this review, we focus on indications for vv-ECMO, components of the circuit, and management of patients on vv-ECMO. Concepts regarding oxygenation and decarboxylation and how they can be influenced are discussed. Day-to-day management, weaning, and most frequent complications are covered in light of the recent literature.

## 1. Introduction

Severe respiratory insufficiency refractory to conventional rescue strategies such as prone positioning requires a degree of pulmonary support not obtainable by mechanical ventilation. Venovenous extracorporeal membrane oxygenation (vv-ECMO) can supply sufficient pulmonary support when gas exchange is severely compromised and presents an ultimate option in problematic cases. However, indications for vv-ECMO are not unequivocal and it is highly invasive, presenting several specific problems and intellectual concepts that require understanding in caring for these patients. This review will focus on these issues and supply the reader with up-to-date knowledge on these unique challenges.

Several other options are available for partial, pumpless lung support or decarboxylation as the primary goal, but they are beyond the scope of this paper. Furthermore, we will discuss vv-ECMO only in relation to adult patients.

Extracorporeal oxygenation has a long history, clinically starting from the first cardiopulmonary bypass for cardiothoracic surgery in 1953 by Gibbon with a vertical screen oxygenator, to development of membrane oxygenation in cardiothoracic surgery in 1969 by Dorson and colleagues. This technique was later used at the bedside instead of the operating theatre for cardiac or respiratory support in several case reports. Through development of better materials and miniaturization, patient outcomes and utilization of the technique in respiratory failure have increased. In particular, the H1N1 epidemic in 2009 has witnessed a surge in vv-ECMO use, with the Extracorporeal Life Support Organization (ELSO) reporting a rise from 100 cases/year from 1996 to 2007 to 480–846 cases/year from 2009 to 2012 [1]. The ELSO is an international nonprofit consortium of health care institutions dedicated to novel therapies of failing organ systems, and its primary mission is maintaining worldwide registry of ECMO in the active ELSO centers.

## 2. Indications

Since vv-ECMO is highly invasive and associated with numerous potential complications, its use should only be considered in patients with a high probability of death with conventional treatment. In the guidelines of the ELSO, several indications for vv-ECMO are given, including hypoxic failure with a mortality risk of 80% or greater (see the list below). This is defined as PaO_2_/FiO_2_ < 100 on FiO_2_ > 90% and/or a Murray score ≥ 3 despite optimal care for 6 hours or more. Hypercapnic failure during lung protective ventilation, which might also be served by smaller decarboxylation devices, and severe air leak are also possible indications. Avoiding intubation in a patient as bridge to lung transplantation is an emerging indication for vv-ECMO, possibly promoting ambulation and decreasing deconditioning [2]. An overview of randomized or propensity matched studies investigating vv-ECMO from 2000 to 2015 reveals various indications being used in clinical practice (Table 1).

*Indications for vv-ECMO.* The indications include the following:

1. Reversible hypoxic respiratory failure when the risk of mortality is 80% or greater.
2. Reversible CO_2_ retention on mechanical ventilation despite high plateau pressures.
3. Severe air leak syndromes.
4. Need for intubation in a patient on lung transplant list.

The Murray Lung Injury Score (LIS), which stems from 1988, was developed as a definition for mild-moderate lung injury (LIS 0.1–2.5) and severe lung injury/ARDS (>2.5) (Table 2) [3]. Usefulness of this score in the era of the revised Berlin criteria for ARDS is questionable. Furthermore, in a 550-patient cohort Berlin stages and LIS score were highly correlated, but the predictive value of both for mortality was limited (AUC 0.58 for LIS and 0.60 for Berlin definition) [4].

In patients where vv-ECMO might be indicated, consideration must be given to other rescue therapies such as prone positioning, recruitment maneuvers, inhaled NO (iNO), and high frequency oscillatory ventilation (HFOV). Survival benefit has been shown in a prospective, multicenter randomized clinical trial for prone positioning [5] and several meta-analyses [6, 7] but not for iNO [8], HFOV [9, 10], or recruitment maneuvers [11].

vv-ECMO has not been unequivocally shown to increase survival. In the only randomized clinical trial to date, patients referred to a vv-ECMO center had significantly improved survival without severe disability. However, only 75% of the transferred patients actually received vv-ECMO [12]. Research groups around the globe report survival rates of 77–79% during the influenza A (H1N1) epidemic in patient groups where conventional scores such as the APACHE and SOFA score predicted much higher mortality [13, 14]. An overview of survival in propensity matched studies reveals overall better survival with vv-ECMO (Table 1). However, propensity matching is dependent on matching criteria and these are not uniform throughout the studies. Pham et al. evidence this, showing nonsignificant and significant survival advantage using different matching criteria [15].

Several outcome scores have been deduced from retrospective cohorts, but none have prospectively been validated [16–19]. Furthermore, these have been developed on cohorts already receiving vv-ECMO and are thus of limited use for predicting outcome before instituting vv-ECMO. These scores indicate better survival in patients of younger age (<45), with lower SOFA/APACHE scores, with a diagnosis of influenza, with shorter duration of mechanical ventilation prior to vv-ECMO, with lower BMI (<30), with nonimmunocompromised state, and being ventilated in the prone position prior to vv-ECMO. In general, the scores show us that sicker patients have worse outcomes, even on vv-ECMO.

## 3. Contraindications

The ELSO guidelines state no absolute contraindications for vv-ECMO since each patient is considered as a candidate individually with respect to risks and benefits. Careful consideration is needed in patients with a dismal prognosis despite successful vv-ECMO due to severe comorbidities, older age (limited evidence of benefit in patients >65 years), multiorgan failure, intracranial bleeding, mechanical ventilation for >7 days at high settings (FiO_2_ > 90%, P-plateau > 30 mmHg), or major pharmacological immunosuppression (absolute neutrophil count < 400/mm^3^).

## 4. Team-Driven Decision

Since indications and contraindications are not set in stone and careful consideration is needed, we believe it is essential to approach the decision to institute vv-ECMO with a multidisciplinary team.

## 5. vv-ECMO Components and Key Concepts

**Figure 1:**
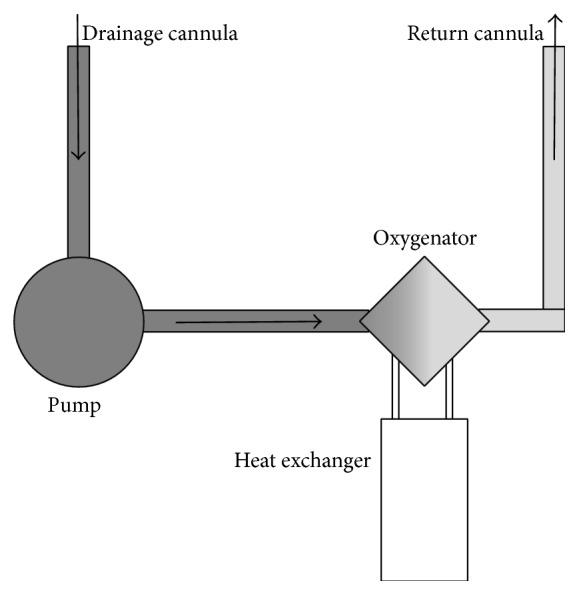
Basic vv-ECMO setup.

The vv-ECMO circuit mainly comprises cannulae, tubing, pump, oxygenator, and a heat exchanger. The drainage cannula removes blood from the patient, mainly driven by gravity funneling. This deoxygenated blood is pumped through the oxygenator and returns, temperature controlled, to the patient through the return cannula (Figure 1).

Different components of the ECMO circuit are connected by polyvinylchloride tubing and connectors. The tubing is a potential source for thrombus formation and activates inflammatory processes via the complement pathway [20, 21]. Bioactive coating, most often heparin, is used to minimize the effects of blood-surface interaction [22, 23].

### 5.1. Cannulation

**Figure 2:**
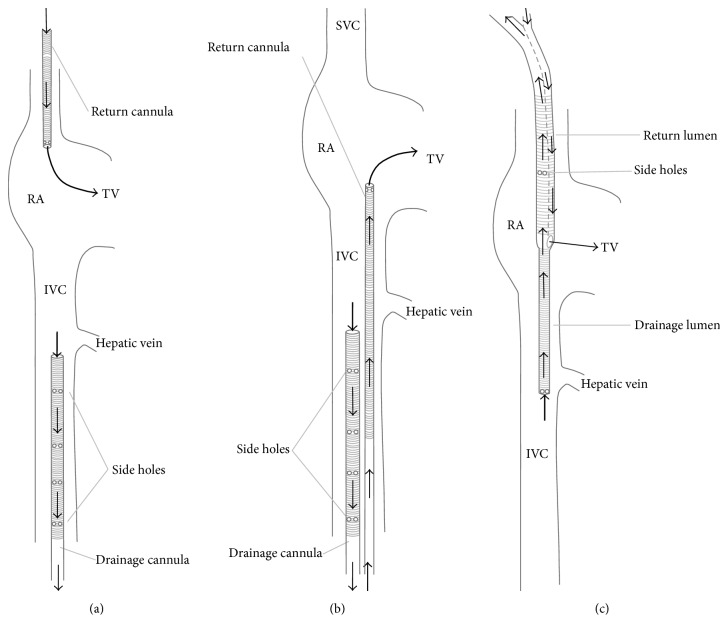
Cannulation options in vv-ECMO. (a) Femorojugular configuration. (b) Femorofemoral configuration. (c) Dual-lumen cannula. SVC: superior vena cava. IVC: inferior vena cava. RA: right atrium. TV: tricuspid valve. Adapted from Sidebotham et al. [38].

Cannulation in vv-ECMO is dual or single site (Figure 2). Dual site cannulation utilizes the internal jugular and/or femoral vein. The femorojugular approach consists of a long drainage cannula inserted through the femoral vein and advanced into inferior vena cava (IVC) with a shorter return cannula inserted into the internal jugular vein (Figure 2(a)) [24, 25]. The second type of dual cannulation, less often utilized, is the femorofemoral cannulation (Figure 2(b)). Major disadvantages of any dual site approach include increased rate of recirculation, insertion site bleeding, infection, immobilization, clot formation, and accidental dislodgement [26]. A single site bicaval dual-lumen cannula is available for establishing respiratory support by accessing the right internal jugular vein (Figure 2(c)) [27]. The cannula simultaneously drains blood from the superior vena cava (SVC) and IVC, while the oxygenated blood is returned into the right atrium via the infusion port pointing towards the tricuspid valve. Benefits of this cannulation are a reduced risk of insertion site bleeding, thrombosis, infection, and accidental displacement of the cannula while facilitating patient mobilization [26]. Some recirculation, to a limited extent, still occurs as a part of design limitations.

Cannula diameter, length, material, and amount of drainage holes will determine the maximal achievable flows and therefore the maximal support that can be delivered. For single lumen setup, sizes from 22–30 French are used for the longer drainage cannula and 15–23 French for the shorter return cannula. Choices in size can be based on (echocardiographic) estimation of vessel diameter or the desired flow. Maximal achievable flows under ideal circumstances are reported in the manufacturer documentation. For the double-lumen cannula 27 or 31 French is recommended to achieve adequate flows to support oxygenation.

### 5.2. Pump and Monitoring

The pump performs two key functions. Firstly, it propels the blood through the oxygenator and returns it back to the patient. Secondly it enhances the venous outflow, mainly driven by gravity funneling, into the vv-ECMO circuit. Two types of pumps are used.

Roller pumps propel blood through compression of the tubing. They are not preload dependent and can ensure constant blood flow. In case of low preload or high afterload, excessive pressure can be generated, damaging tubing and blood. A small venous bladder as reservoir and servo-regulation slowing or stopping the pump with excessive negative pressures can provide a certain safeguard against this problem; however, excessive infusion pressure with risk of blowout remains.

Centrifugal pumps, the most popular option, consist of a magnetically driven impeller that is set in a spiral housing. By rotating rapidly, the impeller creates a pressure gradient. Centrifugal pumps are volume dependent and a set flow cannot be guaranteed. However, this is safeguarded against excessive pressures damaging tubing. Other advantages include a smaller pump and better longevity due to less wear and tear.

In both types of pumps, in-line pressure monitors safeguard preload and afterload. Other monitoring devices included in typical vv-ECMO are saturation sensors for the mixed venous saturation, arterial (return) saturation, and pre- and postoxygenator pressures to estimate pressure drop in the oxygenator. With increasing pressure drop, resulting from clot and fibrin deposition, efficiency of the oxygenator will reduce and mandate an oxygenator exchange.

### 5.3. Recirculation

Recirculation is described as a fraction of oxygenated blood that bypasses the systemic circulation. After reinfusion, the recirculating blood is directly suctioned back into the vv-ECMO system. Recirculation negatively impacts the total amount of systemic blood that is oxygenated and leads to inadequate lung assist, which undermines the very purpose of support.

The extent of recirculation is directly correlated to factors such as cannula type, size, and positioning, pump speed, and blood flows [28]. Additionally, the anatomy of the patient may also influence the dynamics of flow. For instance, rotation of the neck or assuming an upright position from supine position affects the orientation of cannulae, thereby affecting recirculation [29].

Proximity of the reinfusion and drainage ports will have a direct impact on the amount of recirculation, with a higher percentage of recirculation when the two ports are in closer proximity [30]. In dual site cannulation, the distance between the return and drainage cannulae should be at least 10–13 cm [31]. In single site cannulation, the distance between the ports is fixed, but placement of the cannula itself and the surrounding anatomy will influence the amount of recirculation. The positioning of the cannula can be verified using transesophageal echocardiography, if necessary combined with saline injection [26] or fluoroscopy [27]. The application of an ultrasound dilution technique to rapidly quantify the recirculation has been reported [32, 33]. This technique measures changes in fluid density by means of an ultrasound beam through the tubing of the circuit. This approach, described as a simple technique, could further aid in quantifying the recirculation.

### 5.4. Oxygenators

Oxygenators effectively function like a native lung by exchanging gases. The deoxygenated blood, propelled through the pump, enters the oxygenator through the inlet port, undergoes gas exchange, and exits through the outlet port as oxygenated and decarboxylated blood.

#### 5.4.1. Oxygenation

Oxygen transport occurs across the oxygenator membrane. The diffusion of gas is concentration dependent; a larger oxygen gradient promotes better diffusion. A larger surface area also promotes better oxygenation.

In addition to vv-ECMO circuit factors, certain patient related factors play an important role in determining oxygenation such as cardiac output, hemoglobin content, and tissue uptake (as indicated by venous oxygen saturation). The degree of oxygenation rendered through vv-ECMO is directly related to the amount of blood passing through the membrane rather than sweep gas flows. The blood flow required to achieve acceptable arterial oxygenation is usually between 3 and 6 L/minute [31, 34].

While on support the artificial lung is placed in series with the native lung. The improvement in oxygenation will increase the shunt fraction in the native lung due to loss of hypoxic vasoconstriction [35], further lowering the proportion of pulmonary gas exchange in addition to the underlying disease. Nonetheless, the vv-ECMO system is capable of supplying the systemic oxygenation demands of the patient.

#### 5.4.2. Decarboxylation

CO_2_ removal is largely dependent on sweep gas flows across the artificial lung. A relatively low blood flow with high sweep gas flow is sufficient to remove up to 50% of CO_2_ produced by a patient [36, 37], as CO_2_ diffuses 20-fold faster than O_2_. Since CO_2_ removal is more efficient even at low blood flows, it is relatively easier to maintain eucapnic situation than oxygenation in severe ARDS.

CO_2_ exchange is also influenced by surface area and the thickness of the membrane. Therefore, any malfunction of the artificial lung first and foremost affects the CO_2_ transfer. This is reflected by postmembrane increase in pCO_2_, a sensitive indicator for possible loss of membrane function. Condensation of the membrane with vapor or blood in the gas part of the membrane affects ventilation, primarily CO_2_. In such circumstance, sweep gas flows are increased temporarily for less than a minute, while ensuring that the pressure gradient between the gas and blood phases of the membranes is not increased, forcing the condensed vapor to exit the oxygenator [38].

## 6. The Patient on vv-ECMO

### 6.1. Oxygenation during vv-ECMO

Although the ELSO recommends a SaO_2_ target of >80%, there is no general accepted goal of SaO_2_ during ARDS and/or vv-ECMO. A SaO_2_ target of higher than 88% seems reasonable [39], but lower oxygen saturation can be well tolerated by the patient.

Not all venous return is drained by vv-ECMO. This remaining part is “shunted” past the vv-ECMO circuit and undergoes gas exchange in the compromised native lung. This results in a mixed arterial saturation compromising oxygenated vv-ECMO blood and poorly oxygenated “shunted” blood. Since venous return is equivalent to cardiac output, the difference between ECMO flow and cardiac output determines this “shunt fraction.” SaO_2_ of >88–90% is guaranteed with ECMO flow to cardiac output fraction (*Q*_ECMO_/*Q*_CO_) of 0.6, assuming little recirculation, even with absent pulmonary function [31]. In hypoxemic patients with adequate *Q*_ECMO_ but increased *Q*_CO_, resulting in *Q*_ECMO_/*Q*_CO_ below 0.6, interventions to lower cardiac output may be considered. Insufficient analgesia and shivering should be managed first to reduce cardiac output. If high cardiac output persists mild hypothermia or a short acting selective beta-1 blocker may be applied, temperature can be decreased easily via the heat exchanger. Lowering body temperature to 34 degrees has been used successfully in patients with a hyperdynamic circulation [40].

If hypothermia is contraindicated, esmolol, an intravenous selective beta-1 blocker with a short half-life of 9 minutes, can decrease cardiac output in tachycardic, septic patients [41]. In a randomized controlled trial with 77 patients [42], esmolol reduced heart rate in patients with septic shock without adverse events. Monitoring of cardiac output and systemic perfusion is advised to guard against excessive lowering of the cardiac output.

Switch to an alternative mode of cannulation is also a consideration if rescue strategies (prone positioning) and interventions are not successful in restoring SaO_2_ >80%. In refractory hypoxia or with development of cardiac failure, a switch to venoarterial cannulation is an option. This allows hyperoxygenation of the arterial blood but introduces the problem of hypoxic blood being pumped by the heart. This will result in an aortic zone with hypoxic blood originating from the failing lungs depending on the level of cannulation (femoral versus proximal), the ECMO flow, and the cardiac output. This is called the “harlequin syndrome,” with catastrophic outcome if the watershed area is beyond aortic arch, resulting in cerebral hypoxia.

### 6.2. Ventilator Management

ECMO can facilitate lung protective ventilation not achievable with conventional mechanical ventilation in case of severe lung injury. However, optimal ventilator settings for patients undergoing ECMO are unknown and there are no evidence-based guidelines available. In patients with ARDS, ventilation with low tidal volumes of 6 mL/kg predicted body weight reduces mortality [43]. Many patients supported by ECMO are presumed to have ARDS, and minimizing ventilator induced lung injury (VILI) by lowering inspiratory pressures seems mandatory. A systematic review focusing on studies describing ventilation practices during ECLS (49 studies, 2042 patients) showed reduced mortality using “ultra” lung protective settings with tidal volumes <4 mL/kg and plateau pressures <30 cm H_2_O [44].

Optimal level of PEEP in ECMO patients is unclear. Higher levels of PEEP might accelerate lung healing by promoting lung recruitment [45] and in a retrospective, multicenter study higher levels of PEEP (>12 cm H_2_O) during the first 3 days of support were associated with better survival [46].

The ELSO guidelines recommend “rest settings” with plateau inspiratory pressure below 25 cm H_2_O and low FiO_2_ (<30%). Positive end expiratory pressure (PEEP) can be set at values between 5 and 15 cm H_2_O. In the only randomized control trial concerning vv-ECMO ventilator settings used were plateau inspiratory pressure 20–25 cm H_2_O, PEEP 10–15 cm H_2_O with respiratory rate of 10, and FiO_2_ of 30% [12].

In case of refractory pulmonary failure and inadequate support through vv-ECMO, prone positioning is a viable rescue option. Prone positioning is possible under vv-ECMO and risk of accidental decannulation seems limited [47]. In a series of 17 patients prone positioning during vv-ECMO improved oxygenation and respiratory system compliance [48].

Patients on vv-ECMO frequently need mechanical ventilation for a prolonged period of time and early tracheostomy should be considered. Tracheostomy provides less discomfort, easier mobilization, and decreased sedation compared to oral-tracheal intubated patients [49]. Assessment of percutaneous tracheostomy in 118 ECMO patients, with short interruption of anticoagulation, indicates that it is a safe technique when performed by experienced physicians [50].

### 6.3. Fluid/Transfusion Management and Renal Replacement Therapy

Despite difficulty in the initial phase after ECMO initiation (due to capillary leak and/or sepsis), a conservative fluid-management strategy is warranted in ECMO patients [51]. After initial stabilization (usually 12–24 hours), diuretics can be instituted to return and maintain extracellular fluid volume to normal.

In patients with acute kidney injury (AKI), fluid imbalance and electrolyte disturbances may mandate institution of continuous renal replacement therapy (CRRT). Studies using the RIFLE (risk, injury, failure, loss, end stage renal failure) classification showed a 70% incidence of AKI in ECMO patients, which was associated with worse prognosis [52]. CRRT can be combined with ECMO by using an independent vascular access or introduction of a CRRT device parallel into the ECMO circuit [53].

Transfusion management is an area with little guidance in the literature. In general, there is data favoring a restrictive transfusion regime in the intensive care unit. In vv-ECMO patients, there is also data indicating the safety of a restrictive regime, targeting a hemoglobin level of 7–9 g/dL (4.3–5.5 mmol/L) [54, 55]. In our institution, a transfusion trigger of ±10 g/dL (6.0 mmol/L) is adapted according to transfusion needs and clinical situation. We maintain a platelet count of >80.000/mL and consider lowering the transfusion threshold if there are no bleeding complications.

### 6.4. Sedation and Mobilization

During cannulation and the initial period of ECMO therapy (first 12–24 hours), patients may require deep sedation and rarely muscle relaxation for patient comfort and preventing complications such as air embolism caused by spontaneous breathing or dislocation during cannulation. If the patient's condition improves, sedation should be stopped or minimized to allow neurologic evaluation [39]. Sedation and analgesia should be titrated dependent on the patient's anxiety or discomfort. In general, minimal sedation is to be preferred to allow for mobilization and physiotherapy. Despite the use of newer components in ECMO circuits, “medication loss” by adsorption is still significant for drugs like fentanyl and midazolam and reductions up to 50% are reported [56].

Patients should be as mobile as possible depending on primary condition and cannulation configuration. Early mobilization is feasible and safe, even with femoral cannulation [57, 58].

### 6.5. Anticoagulation

#### 6.5.1. Heparinisation

Blood contact with nonendothelialised surfaces results in activation of the clotting system with deposition of fibrinogen, clotting factors, and platelets. This results in a consumptive coagulopathy and thrombocytopenia. Modern circuits for vv-ECMO are coated to improve biocompatibility with the aim of reducing this effect. Despite this formation of thrombi in the circuit, consequent bleeding is still a frequent complication, sometimes necessitating exchanging parts of the vv-ECMO circuit [59].

Heparin is historically used as the primary anticoagulation therapy on vv-ECMO. Heparin binds with antithrombin III (AT3) and potentiates its function, leading to increased inactivation of thrombin. Although originally administered as a fixed dose infusion, heparin can currently be monitored with several coagulation tests. Activated clotting time (ACT), activated partial thromboplastin time (aPTT), anti-Xa activity (aXa), and thromboelastography (TEG)/rotational thromboelastometry (ROTEM) have all been used for monitoring heparin dosage. Many centers use the ACT or aPTT as a guide and some evidence suggests better correlation between heparin dosing and aPTT values as opposed to ACT [60, 61]. In our institution, we use the activated partial thromboplastin time (aPTT), aiming for 1.5–2.0x its normal value.

Heparin requires sufficient AT3 for its effect. Periodic monitoring of the AT3 levels, especially when high dosages of heparin are needed, is advised. Levels should be maintained in the normal range (80–120%).

#### 6.5.2. Acetylsalicylic Acid

Despite heparin use, there can be extensive build-up of clotting deposits necessitating exchange of the oxygenator due to reduced gas exchange capacity. The concomitant use of low-dose acetylsalicylic acid and heparin has been described as a method to lengthen oxygenator time, without apparent increased bleeding risk [62, 63].

### 6.6. Temperature and Infection

Cardiopulmonary bypass and ECMO circuits induce inflammation and systemic inflammatory response syndrome by activating complement system [64]. This response may lead to increased vascular permeability and endothelial dysfunction with capillary leak syndrome necessitating vasopressor treatment and expansive volume loading.

Nosocomial infection risk is high in ECMO patients and related to length of ECMO run, age, and immunosuppression [65]. Blood stream infections and ventilator-associated pneumonia are the most common infections [66]. ECMO cannula infection rate was 10% in an observational study in 2009 [13]. Clinical signs and symptoms associated with infection, in particular fever, may not be recognized in patients on ECMO because temperature can be maintained at any level by adjusting the temperature of the water bath. Incidences of infection during ECMO treatment range widely. A large multicenter database analyzing 1473 patients on ECMO reported infections in 17% of survivors and 28% of nonsurvivors [67]. Difference between ECMO and patient temperature, changes in hemodynamics, and purulent secretions in combination with elevated biomarkers of infection like C reactive protein should raise suspicion of new infection.

Prophylactic antibiotics are not recommended simply because a patient is on ECMO. In case of suspected infection, broad-spectrum antibiotics should be administered early until the results of microbiological cultures become known.

### 6.7. Complications

#### 6.7.1. Bleeding

Bleeding is the most common complication in ECMO because of systemic anticoagulation, thrombocytopenia, and thrombocytopathy. Any routine procedure such as endotracheal suctioning or nasogastric tube positioning and diagnostic procedures such as transesophageal echocardiography can lead to uncontrollable bleeding and therefore should be performed with caution.

The most devastating bleeding complication is intracranial hemorrhage that, according to the ELSO registry, occurs in 4% of vv-ECMO patients with 21% survival rate. Few data predict the incidence of intracranial hemorrhage but prevention of renal failure and aggressive correction of thrombocytopenia may help lower the risk [68]. Duration of ECMO support was not an independent risk factor. Bleeding at cannulation sites is reported in 17% of ECMO patients in the ELSO registry.

In case of bleeding, coagulation should be normalized as much as possible. Correction of thrombocyte and erythrocyte levels, pH, and temperature can be required and local treatment of the bleeding is instituted. In our practice, ROTEM is used for evaluation and rapid correction of coagulation. Targeting lower aPTT (1.0–1.5) and even cessation of heparin can be necessary. Numerous case reports and case series indicate that vv-ECMO can be run without heparin for a certain time in selected cases [69–71]. Tranexamic acid, an antifibrinolyticum, can also be considered [72]. However, any intervention based at minimizing bleeding can aggravate the risk of thrombosis.

#### 6.7.2. Excessive Pump Suction

When insufficient venous return is available to sustain pump flow, a “suck-down” may occur. This usually causes inlet pressures to drop well below −100 mmHg. The vessel wall can be sucked into the access ports of the cannula, obstructing flow into the pump and damaging the vessel. The level of support can drop dramatically, and erythrocytes are damaged leading to hemolysis. Furthermore, there is risk of air embolism due to degassing [73].

Pump speed should immediately be reduced to acceptable suction pressures and cannula position should be examined; an increased caudal position or kinking of the drainage cannula may be the cause of the problem. Secondary causes include hypovolemia, increased abdominal pressure, and cardiac tamponade or pneumothorax [74]. If these causes are excluded and drainage problems continue to occur, a second drainage cannula can resolve suction problems.

#### 6.7.3. Air Embolism

Air embolism may occur due to several causes: inadvertent entry of atmospheric air into the circuit, degassing, or elevated sweep gas pressure. Tubing connections and three-way valves are risk sites for introduction of air. Increasing the complexity of the circuit will also increase the risk for air embolism due to more connections and valves. Degassing can occur if suction pressures reach critical levels, resulting in gaseous microemboli [73]. If sweep gas pressure exceeds blood pressure, air bubbles may pass through the membrane. Prevention consists of keeping the membrane lung below the level of the patient and maintaining higher blood side than gas side pressure by a pressure pop-off valve or pressure servo-regulation control in the sweep gas supply.

In case of a large air embolism heading towards the patient, the arterial cannula should be clamped close to the patient, the pump turned off, and the embolism removed. Although the risks of air embolism appear smaller during vv-ECMO, due to the filtering function of the lungs, passage through a patent foramen ovale or cardiac standstill due to air lock is possible.

#### 6.7.4. HIT

Heparin-induced thrombocytopenia (HIT) is a feared complication of exposure to heparin, with an estimated incidence of 1-2% in postcardiac surgery patients [75]. HIT results from an antibody formed against platelet factor 4 (PF4) combined with heparin. Two types of HIT have been identified: type I is a mild and transient drop in thrombocytes and there is a clinical significant syndrome with thrombocytopenia in type II. Both bleeding, due to coagulopathy, and thrombosis, due to platelet activation, are the result of this immunologic complication.

The diagnosis can be difficult due to the multiple factors inciting thrombocytopenia in the intensive care unit patient [76]. The most commonly used test for HIT is an ELISA-based test with a high rate of false positives. The more specific functional tests (serotonin release assay or heparin-induced platelet aggregation assay) are not available in all hospitals.

Upon diagnosing or strongly suspecting a diagnosis of HIT, it is imperative that heparin should be substituted for a different anticoagulant. There are numerous case reports and series for argatroban [77, 78], bivalirudin [79], danaparoid [80], lepirudin [81, 82], and fondaparinux [83]. Although exchange of the ECMO system is often necessary due to thrombosis, the heparin-coating does not appear to cause or perpetuate HIT [84].

#### 6.7.5. Thrombosis in the ECMO System

Although the technique for the different parts of the ECMO system has progressed substantially, it is still not ideal for long-term durability. In particular, the oxygenator is at risk for fibrin deposits and formation of clots. The efficiency of the oxygenator is decreased with more extensive deposits, resulting in reduced gas exchange capacity and increased resistance to flow [85]. The only remedy is exchange of the involved component. D-dimers, in the absence of other explaining pathologies, can predict clot volume and oxygenator exchange [86, 87]. As mentioned previously, acetylsalicylic acid can be used as a treatment to reduce thrombocyte deposits in the oxygenator in addition to adequately dosed heparin.

### 6.8. Interfacility ECMO Transportation

Transporting critically ill patients is a high-risk procedure with a significant rate of critical events [88–93]. Deterioration of the patient's condition during or shortly after transport can occur due to the absence of adequate equipment, technical failure of the equipment, insufficient treatment during transport, or finally the natural course of the disease. The complexity in transporting patients while being supported with ECMO therapy reveals numerous possibilities for life threatening complications. Concerning transport of critically ill patients with ECMO, the amount of adverse events varies widely in the literature (0%–42%). The existing literature consists mainly of retrospective single center case series with patients numbers from 10 to 282 without consistent definition of adverse events. Furthermore, there is a case mix of patients being cannulated off center by an ECMO retrieval team and those being transported after cannulation in an ECMO center [94–99]. From these reports, it appears that transporting critically ill patients on vv-ECMO can be performed without a significant rate of life threatening complications if (1) these transports are performed by specialized retrieval teams, trained in both ECMO therapy and interfacility transport, (2) adequate equipment is provided, and (3) the transport vehicle offers sufficient space to guarantee patient and team safety during transport.

A position paper, by the International ECMO network regarding organization of extracorporeal membrane oxygenation, advises the creation of a mobile ECMO team by tertiary expert ECMO centers [100].

In our institution, the ECMO retrieval/transport team consists of 1-2 intensivists (depending on eventual off-site cannulation), an intensive care nurse, and a perfusionist.

### 6.9. Weaning

Weaning from vv-ECMO is more or less a process of trial and error and no randomized controlled trials have evaluated different strategies. Nevertheless, a weaning strategy or protocol is highly recommended. Strategies may differ and depend on cannulation type, time course, and reversibility of the primary disease.

In case of a rapid reversible course of the primary disease, the simplest strategy is decreasing sweep gas flow and oxygen fraction in the oxygenator during at least 2 hours with maintained pump flow. In this way, no adjustments are necessary regarding anticoagulation and blood flow can be maintained with minimal coagulation risks. In case of failure vv-ECMO treatment is easily resumed. It is important to monitor SaO_2_, pCO_2_, respiratory rate, and minute volume and adjust the mechanical ventilator if necessary. Discontinuation of vv-ECMO can be considered when the O_2_ fraction in the oxygenator is 21% and the sweep gas flow has been minimized with acceptable mechanical ventilation settings.

In case of more complicated weaning, a gradual decrease in support may be useful, analogous to weaning from a mechanical ventilator after a prolonged period. This may allow a progressive adjustment of the pulmonary function and, if necessary, a metabolic adjustment of the pH in case of developing permissive hypercapnia.

Weaning from the mechanical ventilator while continuing vv-ECMO is also a possibility. In particular with single site cannulation the patient can become ambulant earlier, minimizing deconditioning. A case-by-case consideration is necessary to evaluate primary weaning from mechanical ventilation or vv-ECMO support. In particular as a bridge to transplantation this ambulatory, not-intubated strategy is interesting [101–105]. A recent systematic review found no compelling evidence as of yet for vv-ECMO as an alternative bridging strategy compared to mechanical ventilation while waiting for lung transplantation. However, one-year survival was comparable for vv-ECMO bridged and mechanical ventilated patients, challenging the contemporary view of vv-ECMO as a contraindication for lung transplantation [106].

### 6.10. Futility

Futility can be described if vv-ECMO is no longer meeting its intended goal as bridge to transplantation or recovery because both goals are no longer viable. In case of futility and cessation of vv-ECMO support, a multidisciplinary discussion is imperative to ensure that all possible options have been exhausted. This situation is ethically complex and sensitive and there are case reports of support periods over 100 days with ultimately successful weaning [107, 108]. No further escalation of therapy, including replacement of ECMO parts with a limited durability such as the oxygenator, may represent a reasonable option in a futile scenario [109].

### 6.11. Team Approach/Centralization of Care

As stated previously, the decision regarding instituting vv-ECMO should be approached in a multidisciplinary team. The subsequent intensive care management should also be characterized as a cooperative and multidisciplinary setting. Given the various issues and complications during vv-ECMO, it can be necessary to include specialists from various areas. This raises the question of centralization of ECMO care, since sufficient case volume is needed to gain experience and maintain competence. This is an area of debate in which national and international guidelines need to be developed. A recent position statement argued a case volume of at least 12 vv-ECMO cases per year [100]. This statement represents the consensus opinion of a large group of healthcare workers with expertise in vv-ECMO.

## 7. Future Directions

ECMO for respiratory support has progressed significantly since its first reported application [110] over 40 years ago. Miniaturization, better biocompatibility, improved designs, and recent H1N1 epidemics have rekindled interest in vv-ECMO for adult patients. Currently, given the lack of robust evidence in favor of vv-ECMO treatment, it is a modality restricted to the most severe cases as a last resort. More evidence is forthcoming to evaluate its role as an earlier strategy in severe ARDS to limit VILI [111].

Given the fact that vv-ECMO is highly invasive and can be technically complicated, efforts to improve and simplify components of the treatment are crucial in improving outcomes in the future. One important technological advance in the extracorporeal technology is the miniaturization of the entire ECMO system into a hand-held, portable system with an oxygenator, a pump, and all of the components necessary to provide support [112–114]. Since bleeding is the most feared and lethal complication in ECMO, optimal anticoagulation is also a matter of debate. Knowledge on pharmacodynamics during ECMO treatment will be improved by upcoming trials. Alternatives to heparin [115, 116] and tracking of heparin activity by other methods compared to the aPTT/ACT are areas of interest. Optimal ventilator setting in vv-ECMO is unclear and upcoming trials will hopefully shed light on this topic [117].

## 8. Conclusion

vv-ECMO can be a viable alternative in severe cases of respiratory insufficiency, offering a bail-out option in case other treatments fail. Miniaturization, better biocompatibility, and improved designs bring this treatment within reach for more patients. However, concepts in oxygenation, decarboxylation, and patient management on vv-ECMO merit special attention and expertise. In addition, a multidisciplinary approach is mandatory.

## Figures and Tables

**Table 1:** Randomized or propensity matched studies with vv-ECMO.

Year, author	Study type	Method	Inclusion	ECMO indications	*n*	ECMO duration (days)	Survival
2009, Peek et al. [12]	Multicenter RCT	Randomization to referral ECMO center versus conventional treatment in referring hospital	ECMO indication	18–65 years, reversible respiratory failure + Murray ≥ 3.0 or respiratory acidosis (pH < 7.2)	180 (90 vv-ECMO, 90 conventional)	9	63% (ECMO) versus 47% (conventional) 6-month survival without disability (*p* = 0.03)
2011, Noah et al. [118]	Prospective, multicenter cohort study with propensity matching	2009-2010 Swift database; suspected and confirmed H1N1 in 192 ICUs in the UK	Referral to an ECMO center	18–65 years, reversible respiratory failure + Murray ≥ 3.0 or respiratory acidosis (pH < 7.2)	80 patients referred (69 vv-ECMO) 75 propensity matched ECMO patients	9	76% survival to discharge (ECMO) versus 53% (propensity) (*p* = 0.01)
2013, Pham et al. [15]	Prospective, multicenter cohort study with propensity matching	2009-2010 H1N1 infected patients in 114 participating French ICUs	H1N1 related ARDS treated with ECMO	Not specified	123 ECMO patients (107 vv-ECMO, 16 va-ECMO) 52 propensity matched ECMO patients	11	50% (ECMO) versus 40% (conventional) (*p* = 0.32, NS)
2014, Guirand et al. [119]	Multicenter cohort study	2001–2009 database in 2-level I trauma centers in the US	Acute hypoxic failure (PaO_2_/FiO_2_ < 80 + FiO_2_ > 90% + Murray >3 .0)	16–55 years, PaO_2_/FiO_2_-ratio ≤80, FiO_2_ > 0.9, Murray > 3.0	26 vv-ECMO 17 propensity matched ECMO patients	32	65% (ECMO) versus 24% (conventional) (*p* = 0.01)

**Table 2:** Murray Lung Injury Score.

	0	1	2	3	4
PaO_2_/FiO_2_ on FiO_2_ 100%	300 mmHg	225–299 mmHg	175–224 mmHg	100–174 mmHg	<100 mmHg
Chest X-ray quadrants	Normal	1	2	3	4
PEEP (cmH_2_O)	≤5	6–8	9–11	12–14	≥15
Compliance (mL/cmH_2_O)	≥80	60–79	40–59	20–39	≤19
